# Reliability and Validity of the Athletic Shoulder (ASH) Test Performed Using Portable Isometric-Based Strength Training Device

**DOI:** 10.3390/biology11040577

**Published:** 2022-04-11

**Authors:** Aleksandra Królikowska, Anna Mika, Bartosz Plaskota, Maciej Daszkiewicz, Monika Kentel, Anna Kołcz, Maciej Kentel, Robert Prill, Dorota Diakowska, Paweł Reichert, Artur Stolarczyk, Łukasz Oleksy

**Affiliations:** 1 Ergonomics and Biomedical Monitoring Laboratory, Department of Physiotherapy, Faculty of Health Sciences, Wroclaw Medical University, 50-367 Wroclaw, Poland; bartosz.plaskota@student.umed.wroc.pl (B.P.); maciej.daszkiewicz@student.umed.wroc.pl (M.D.); anna.kolcz@umw.edu.pl (A.K.); 2 Institute of Clinical Rehabilitation, University of Physical Education in Krakow, 31-571 Krakow, Poland; anna.mika@awf.krakow.pl; 3 eMKaMED Medical Center, 53-110 Wroclaw, Poland; monika.kentel@emkamed.com.pl (M.K.); maciej.kentel@emkamed.com.pl (M.K.); 4 Center of Orthopaedics and Traumatology, Brandenburg Medical School, University Hospital Brandenburg/Havel, 14770 Brandenburg, Germany; robert.prill@mhb-fontane.de; 5 Department of Basic Sciences, Faculty of Health Sciences, Wroclaw Medical University, 50-367 Wroclaw, Poland; dorota.diakowska@umw.edu.pl; 6 Department of Trauma Surgery, Faculty of Medicine, Wroclaw Medical University, 50-367 Wroclaw, Poland; pawel.reichert@umw.edu.pl; 7 Orthopaedic and Rehabilitation Department, Medical Faculty, Medical University of Warsaw, 02-091 Warsaw, Poland; artur.stolarczyk@wum.edu.pl (A.S.); loleksy@oleksy-fizjoterapia.pl (Ł.O.); 8 Physiotherapy and Sports Centre, Rzeszow University of Technology, 35-959 Rzeszow, Poland

**Keywords:** Active5, biomedical monitoring, injury prevention, force plates, physiotherapy, rehabilitation, sports medicine, upper limb

## Abstract

**Simple Summary:**

The Athletic Shoulder (ASH) test was developed to quantify force across the shoulder girdle in athletes for diagnosis and monitoring. Initially, this test was performed using force plates. The question remains whether force plates may be replaced with a more feasible tool for field testing, such as an isometric-based strength training device. Hence, the present study determined whether Active5™ may be an alternative to force plates for ASH test purposes. Consequently, the ASH test was performed on different days by the same rater and different raters using Active5™ and K-Force plates. It was also checked whether the test results obtained using various tools correlated with each other. The study indicated that both devices were reliable tools, and the ASH test results obtained with the use of the two devices were largely correlated with each other.

**Abstract:**

The Athletic Shoulder (ASH) test was introduced as a tool for quantifying the ability to produce and transfer force across the shoulder girdle. Whether using the portable isometric-based strength training device Active5™ is a reliable alternative to a gold standard force plate for ASH testing purposes remains unknown; therefore, the present study determined the reliability and validity of Active5™ usage in the ASH test compared to force plates. Fifty-one healthy participants performed the ASH test using Active5™ and K-Force plates in three separate sessions. The maximal force was measured bilaterally in a prone position at three shoulder abduction angles, precisely at 180°, 135°, and 90°. The first rater carried out the first and third sessions, spaced at a one-week interval. A second rater performed the second session. The reliability was assessed using the intraclass correlation coefficient (ICC). The linear Pearson’s correlation coefficient (*r*) calculation was used to determine the relationship between ASH test results using the two devices. The ICC = 0.77–0.99 result indicated good to excellent reliability for Active5™ usage. A high to a very high correlation between the two devices at 180° and 90° was noted (*r* = 0.75–0.95). This data supports the isometric-based strength training device Active5™ as a reliable and valid tool for ASH test performance.

## 1. Introduction

The evaluation of muscle strength constitutes an essential component of shoulder examination. An athlete’s assessment, treatment, and performance enhancement may be significantly facilitated using isokinetic and isoinertial test procedures, which have scientific and clinical rationale for their use in sport and rehabilitation [1,2,3,4]. Since isokinetic and isoinertial testing provide easily interpreted objective data, they are commonly recognized as the gold standard for strength testing. However, because of the expensiveness of isokinetic dynamometers and piezoelectric force plates, these methods are not available to many clinicians and strength and conditioning professionals. Additionally, as an isokinetic dynamometer takes up a lot of space and the examination is time-consuming, it is challenging to implement isokinetics in sports settings. Because of its low price, convenient size, and ease of use, hand-held dynamometry (HHD) has been considered a static alternative to isokinetic dynamometry and the use of force plates in strength measurements [5,6,7]. However, the reliability of HHD remains questionable due to many potential sources of error in measurements, including various warm-up strategies or starting positions for both tester and person being examined, the position of the examined shoulder, stabilization of the tested limb, and stabilization of the measurement device [8,9,10,11]. The HHD is not considered fully reliable, especially in sports, because it is also affected by the strength of the tester, as stronger athletes may require stronger testers to resist the athlete’s push and obtain reliable results [12].

The Athletic Shoulder (ASH) test was developed by Ashworth et al. (2018) as a novel series of long-lever upper body isometric tests conducted with the use of force platforms [13]. It is performed in the prone position with the examined shoulder positioned at three consecutive angles of abduction, precisely 180°, 135°, and 90°. Using force plates for measuring isometric force was introduced as a potential alternative to hand-held dynamometers. As the examined person applies force to a fixed device, the tester’s strength as a potential source of measurement error is eliminated. Once more, Ashworth et al. (2018) highlighted that long-lever isometric tests seem more reasonable than short-lever testing, as they replicate the shoulder muscle contraction required in the tackle position [11,13,14] and therefore have greater potential in shoulder injury prevention [15]. Still, force plates are more expensive than and not as portable as HHD.

Recently, Activbody Inc. released a new device designed to work with apps and games running on Bluetooth-enabled devices, such as smartphones and tablets, named Active5™. It is described as a portable isometric-based strength training device with digital coaching. Active5™ measures the maximal isometric strength and adjusts the difficulty of an exercise individually. Because the device is brand new, there are no examples of its use in functional diagnosis or the effectiveness of its usage as exercise-based therapy support available in the literature to date. Thus, the reliability and validity of strength measurements taken using the device also remain unknown. However, if Active5™ measures force by being pressed with the examined limb or another part of the body, it might constitute a portable alternative for ASH testing purposes to the gold standard force plate. Therefore, this study aimed to determine the reliability and validity of Active5™ usage in the ASH test compared to a force plate.

## 2. Materials and Methods

### 2.1. Ethics

The study was carried out in 2021–2022 in the Ergonomics and Biomedical Monitoring Laboratory and the Department of Trauma at Wroclaw Medical University, Wroclaw, Poland. The study was conducted according to the Declaration of Helsinki’s ethics guidelines and principles. It was approved by the Bioethics Committee at the Medical University of Wroclaw, Wroclaw, Poland (approval number KB-351/2021). All participants received a detailed explanation of the study purpose and the study procedures, and subsequently signed a written informed consent form.

### 2.2. Study Design

The study used a randomized, repeated measure, observational design. First, the two devices were compared in terms of their intrarater and inter-rater reliability in performing the ASH test. Therefore, the variation of data gathered by one rater across two trials (intrarater reliability) and the variation of data collected by two raters who examined the same participants (inter-rater reliability) was analyzed. The agreement on the use of both testing devices was determined. It was also estimated whether any linear correlation between the results of the ASH test using the two devices existed. The data were separately explored for women and men.

### 2.3. Characteristics of Studied Participants

The study was carried out in a group of the first 51 recreational athletes from the university where the study was conducted who voluntarily participated in the study and met all the inclusion criteria. The volunteers were recruited through advertising on university social media. The specific inclusion criteria were as follows: (1) age 18–30 years; (2) free of injury and/or disease in the upper limb(s) and cervical or thoracic spine; (3) not having pain or any other symptoms in the upper limbs or cervical or thoracic spine; (4) not having any systematic disease; (5) frequently participating in sporting activities. The dominant limb was determined to be that normally used for daily activities and writing. Anthropometric characteristics were collected before the tests, including body weight (kg) and height (cm). For data analysis purposes, the participants were divided into women and men.

### 2.4. Measurement Procedure

The participants were tested on three separate occasions. The first and third sessions were carried out by rater number 1 at a one-week interval, while the second was performed by rater number 2 on day one. On the first day, the break between the session carried out by the first rater and the session carried out by the second rater exceeded 90 min. Both raters were well experienced with the equipment and the test protocol. The tests were performed at the same time of day. All the participants were asked to wear sports clothes for the tests. They were also asked to maintain their regular training regimes during the experimental period and not to participate in any vigorous physical activity between the three sessions of performed tests.

**Figure 1:**
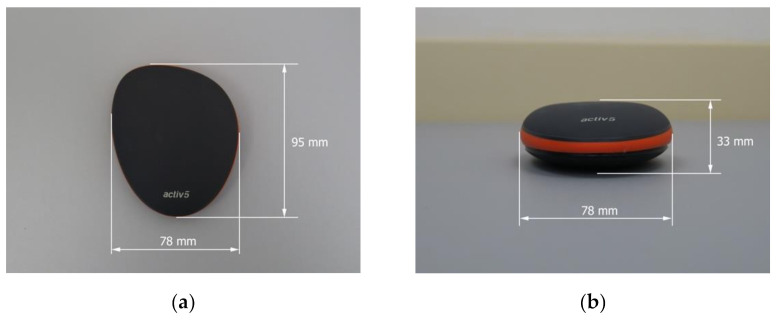
Technical characteristics of portable isometric-based strength training device Active5™: (**a**) Length and width; (**b**) width and height. The weight exceeds 131 g, including the battery.

**Figure 2:**
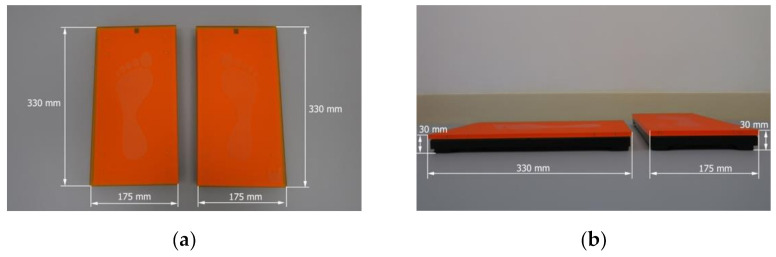
Technical characteristics of K-Force Plates separately for right and left limbs: (**a**) Length and width; (**b**) length, width, and height. The weight exceeds 1600 g.

At each session, the ASH test was performed two times, each time with a different device, namely Active5™ (Activbody, Inc., Shenzhen, China) and K-Force Plates (Kinvent, Montpellier, France). Both devices were Bluetooth-enabled and connected with Active5 (version 1.0.0.; Activbody, Inc., China) and K-Force (version 5.2.1; Kinvent, Montpellier, France) applications, respectively. The applications were installed on a tablet (Galaxy Tab S7 SM-T870, Samsung Electronics Co., Ltd. 129, Samsung-ro, Yeongtong-gu, Suwon-si, Gyeonggi-do, Republic of Korea). Detailed dimensions of Active5™ are presented in Figure 1, and the dimensions of K-Force Plates in Figure 2.

Before the first session measurements, the participants were randomly assigned to start on the Active5™ or K-Force plate with either the dominant or nondominant limb. This order was replicated in session two and session three. After the measurement of one limb with one device was completed, the contralateral limb was tested. After 30 min, the procedure was repeated with the other device.

**Figure 3:**
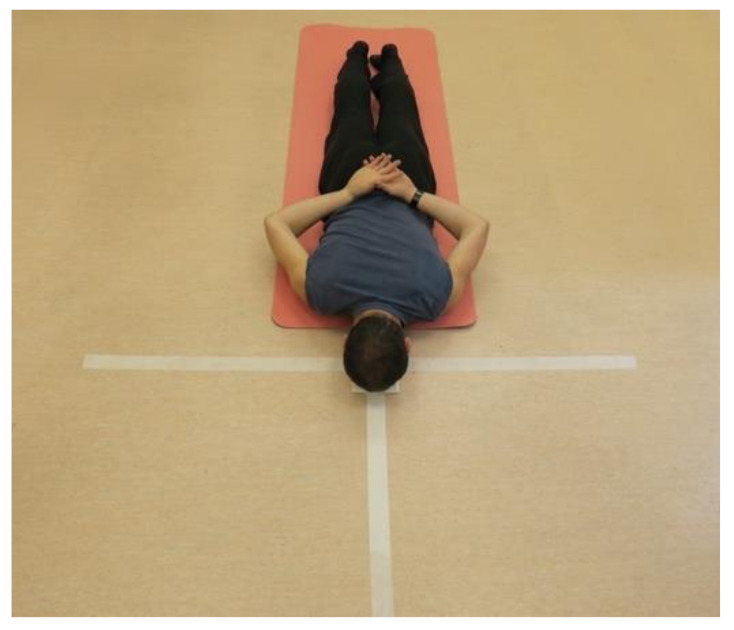
The preparation of the participant before the consecutive series of tests.

The tests with the portable isometric-based training device and force plates were performed with the participant in a prone position on a thin mat laid on the floor, with a neck position standardized using a folded towel of 1 cm height as a forehead support, as presented in Figure 3.

The present research was based on the ASH test methodology introduced by Ashworth et al. (2018) [13]. The participants were familiarized with the test protocol by performing complete tests on separate days before the study. During measurements, standardized verbal commands and consistent verbal encouragement were provided.

A standardized warm-up that consisted of two submaximal efforts in each testing position preceded the series of tests with a given device. During the series of tests, the participant performed a 3 s long maximal isometric contraction against a given measurement device at three shoulder abduction angles, precisely at 180°, 135°, and 90°, also called the I test, Y test, and T test, respectively, as presented in Table 1. The correctness of the angular position of the limb being examined was verified each time with a standard goniometer. The stationary arm of the goniometer was parallel with the trunk, and the movement arm was in line with the midline of the humerus of the studied limb. The axis of the goniometer was positioned slightly below the posterior side of the acromion. The participants were asked to push the device as hard as they could to generate maximal force. Three maximal repetitions were performed at each position with a 20 s long interval, but only the maximal value was taken for further analysis. The hand of the examined limb was placed on a vertical axis force platform or the portable isometric-based training device, and it acted as the primary contact point with a given device. At each position, the forearm was pronated and the elbow fully extended.

### 2.5. Statistical Analysis

SPSS Statistics Version 28.0.1.0 (142) (IBM^®^ SPSS^®^ Statistics, Armonk, NY, USA) and Microsoft Office Excel 365 Personal (Microsoft Corporation, Redmond, WA, USA) were used for the statistical analysis.

The calculation used to choose the number of participants was based on the research of Bujang and Baharum (2017), an article in which the relationship between the interclass correlation coefficient (ICC), statistical power, and the number of participants is established [16]. Considering the necessity for two measurements to be made per participant to separately determine intrarater and inter-rater reliability, set a statistical power of 80%, and establish a minimum ICC of 0.50, the sample for analysis needed to number at least 22. Therefore, the two homogenous groups, including 24 female and 27 male participants, were considered sufficient.

The collected parameter was “maximal force applied against the device”, expressed in kilograms (kg) at three consecutive positions at three separate sessions using both studied devices separately for dominant and nondominant limbs.

The arithmetic mean (x) and standard deviation (SD) were calculated for the studied features. All the studied features were normally distributed according to performed Shapiro–Wilk test.

The relative reliability assessment of each device was based on the intraclass correlation coefficient (ICC) calculation according to the guidelines described by Shrout and Fleiss (1979) [17]. For the intrarater test reliability, a two-way mixed-effects model, single measurement type, and absolute agreement definition were used. The inter-rater test reliability was assessed using ICC calculated using a two-way random-effects model, single rater type, and absolute agreement definition [18]. ICC values less than 0.50 indicate poor reliability, values between 0.50 and 0.75 demonstrate moderate reliability, values between 0.75 and 0.90 indicate good reliability and values greater than 0.90 indicate excellent reliability [18].

The absolute reliability was determined by computing the standard error of measurement (SEM) and coefficient of variation (CV) [19]. The SEM calculated the minimal detectable change (MDC) for each device with the following formula: MDC = SEM × 1.96 × √2. A smaller MDC indicates a more sensitive measurement [20]. Heteroscedasticity was examined using the Bland–Altman method [21].

The linear Pearson’s correlation coefficient (*r*) was calculated to measure the strength and direction of any linear relationships between the obtained values of maximal force (kg) at three consecutive positions in three separate sessions separately for dominant and nondominant limbs. The magnitudes of all of the bivariate associations were classified as negligible (0.00–0.30), low (0.31–0.50), moderate (0.51–0.70), high (0.71–0.90), and very high (0.91–1.00) [22]. Additionally, the coefficient of determination, the *r*-squared (*r*^2^) value, was calculated to give a proportion of variance (fluctuation) of one variable that is predictable from the other variable. The *r*^2^ multiplied by 100 represents the percentage of data points closest to the line of best fit.

The statistical significance was set at *p* < 0.05.

## 3. Results

The female group consisted of 24 people with a mean age of 21.87 ± 1.29 years old, body height 168.77 ± 6.44 cm, and body mass 62.90 ± 6.84 kg. The group of males consisted of 27 people with a mean age of 22.96 ± 2.39 years old, body height of 181.74 + 5.56 cm, and body mass of 81.92 + 12.83 kg.

Based on the ICC results presented in Table 2, it can be stated that intrarater reliability for the group of females was from good to excellent for Active5™ and excellent for K-Force Plates at 180°, 135°, and 90° shoulder abduction.

The inter-rater reliability was excellent for both Active5™ and K-Force Plate devices at 180°, 135°, and 90° of abduction of the shoulder in the female group, as presented in Table 3.

As presented in Table 4, the force values measured using Active5™ in the female group at 180° shoulder abduction represented variation ranging from 28.16 to 29.65% for the dominant limb and 32.29 to 33.89% in the nondominant one.

The force values measured in the same group using K-Force Plates at 180° shoulder abduction represented a comparably wide variation, as the CV ranged from 30.37 to 31.53% for the dominant limb and 34.58 to 38.14% for the nondominant limb (Table 4). Moreover, the values of SEM for the force measured at 180° shoulder abduction were comparable for both studied devices, as presented in Table 4. In addition to similar CV and SEM values, the comparability of the results obtained at 180° shoulder abduction using both studied devices were supported by the high positive correlations presented in Table 5 and Table 6 for dominant and nondominant limbs, respectively.

As presented in Table 7, the CV of force measured using both Active5™ and K-Force Plates at 135° shoulder abduction in the female group was larger than the CV of values of force estimated at 180° shoulder abduction. Additionally, SEM was more prominent than in the measurement at 180°. Both CV and SEM differences between the two studied devices were also reflected in the lack of correlations, as presented in Table 5 and Table 6. However, it should be highlighted that both CV and SEM were smaller in the measurements performed using Active5™ than those using K-Force Plates, indicating a smaller dispersion of data points.

As presented previously in Table 5 and Table 6, the ASH test results using Active5™ at 90° shoulder abduction were highly positively correlated with those obtained using K-Force Plates in both dominant and nondominant upper limbs in females. The CV and SEM of the gathered data were comparable, but not as much as in the measurements performed at 180° shoulder abduction, which are presented in Table 8. It is also worthy of notice that the dispersion of data points was higher for both devices when measuring at 90° than at 180°. Still, the dispersion was smaller for the data gathered using Active5™ (Table 8).

Based on the ICC results presented in Table 9, it can be determined that intrarater reliability in the group of males was from good to excellent for Active5™ at 180°, 135°, and 90° for nondominant shoulder abduction and excellent at 135° for dominant shoulder abduction. K-Force Plates in the group of males represented good to excellent reliability at 180° abduction of the shoulder in the dominant limb. The reliability was excellent for the rest of the measured angles in the dominant limb and for all the measurements in nondominant limbs when using K-Force Plates.

The inter-rater reliability of the force measurements performed using Active5™ in males was from good to excellent at 180° abduction in the dominant shoulder and at 180° and 135° in the nondominant one (Table 10). The reliability was excellent with the dominant shoulder abducted to 135° and 90°. The reliability of the measurements performed using K-Force Plates was good to excellent at a 135° angular position of the nondominant shoulder. The reliability was excellent for the rest of the angular positions in the nondominant shoulder and for all angular positions of the shoulder in the dominant limb.

As presented in Table 11, the force values measured using Active5™ in the male group at 180° shoulder abduction represented variations ranging from 31.65 to 36.98% for the dominant limb and 30.82 to 32.92% in the nondominant one. The data variations gathered using K-Force Plates exceeded 33.25–36.95% and 35.25–37.57% for dominant and nondominant limbs, respectively. The comparable values of CV and SEM of force measured when using both devices at 180° were also reflected in a high positive correlation, as presented in Table 12 and Table 13.

In contrast to the female group, the males presented a smaller dispersion of data points for force measurements performed at 135° shoulder abduction when using K-Force Plates than Active5™ (Table 14). As shown previously in Table 12 and Table 13, no correlation was found between the results of the ASH test performed at 135° using both studied devices.

As presented previously in Table 12 and Table 13, the ASH test results using Active5™ at 90° shoulder abduction were positively correlated from a moderate to a high level with those obtained using K-Force Plates in both dominant and nondominant upper limbs in males. The CV and SEM of the gathered data were comparable, as presented in Table 15; however, the between-devices differences were higher than at 180° but more minor than at 135°.

The upper and lower limits of the agreement of the mean differences between force measurement results were determined using the Bland–Altman plots attached in the Supplemental Material (Supplementary Material S1).

## 4. Discussion

The study aimed to determine the reliability and validity of Active5™ usage in the ASH test compared to a force plate. The two devices were compared in terms of their intrarater and inter-rater reliability in performing the ASH test. Additionally, we examined whether any linear correlation exists between the results of the ASH test conducted using the two devices. The analysis was performed separately for women and men. The results from our study showed that intrarater reliability was good to excellent for Active5™ and was excellent for K-Force plates. Inter-rater reliability was excellent for both devices. It was also determined that the results of the ASH test performed using Active5™ strongly correlated with those obtained using K-Force plates at 180° and 90° shoulder abduction. The results obtained with different devices at 135° should be always compared with considerable caution.

Shoulder injuries are very common among athletes; therefore, appropriate diagnostic methods are of high interest. The ability to quantify shoulder strength quickly and accurately may be especially important during athlete monitoring throughout the season and when making return-to-play decisions [23,24]. The most important consideration in shoulder strength assessment is that the measurement should be performed in a position close to that relating to a shoulder injury [25,26]. The most common mechanism of sports injury is when a resisted force causes the shoulder to be pushed into extremes of range in horizontal abduction or flexion [23,26]. The ASH test, when performed using a straight arm with force delivery at the end of a long lever, allows a condition very like force transfer requirements experienced in sporting actions to be obtained [23,26,27]. It was reported previously that the ASH test performed with a force plate might quantify force production in long-lever positions, defined as the three arm positions, precisely 180°, I test; 135°, Y test, and 90°, T test, which may indicate potential interlimb or post-injury deficits [28].

Isokinetic testing is considered to be the best method for muscle strength assessment [29], but as was underlined, for example, by Impellizzeri et al. (2009), this method does not allow for the evaluation of shoulder strength in similar positions to the mechanisms of injury [15]. Furthermore, isokinetic testing requires expensive equipment, which is very often not accessible for amateur clubs [25,29]. Other methods reported in shoulder force assessment are HHD [8], manual muscle testing [30], sphygmomanometer testing [25,31,32], and force plates [33,34]. The lowest reliability was reported for manual muscle testing [30]; therefore, its utility is limited despite its affordability. Sphygmomanometer testing has been described as a cheap and reliable method of strength assessment for lower limbs [31] and shoulder strength in internal, external rotation, and scaption [35]. Additionally, high correlations were found between the force platform and sphygmomanometer for all three test positions [35].

The reported reliability of upper limb strength measured with HHD in the internal and external rotation was high for the following positions: standing, ICC: 0.92–0.96 [36]; seated, ICC: 0.68–0.99 [37], and in multiple positions, ICC: 0.93–0.99 [8]. The major drawback of HHD testing is the short lever arm of strength measured. Therefore, some authors have suggested that this test is vulnerable to errors, which may be the reason for its lower reliability. It was suggested by Holt et al. (2016) that the higher forces produced by stronger athletes may induce tester–athlete strength imbalances and therefore reduce HHD test reliability [38]. They postulated a stable force platform to be more appropriate for these populations [8,38]. The force platform was described as a valid tool for assessing isometric strength [33]; if the subject applies force to a fixed device, the tester strength as a potential source of measurement error is eliminated. Because HHD is a portable device, highly accessible, and easy to use, it is probably the most popular upper limb strength assessment method, despite its weaknesses.

Force plates have been considered much more portable and, depending on their hardware, much cheaper than isokinetic dynamometers and more reliable than a sphygmomanometer or HHD for upper limb and shoulder girdle strength testing. Additionally, Ashworth et al. (2018) found excellent reliability (ICC = 0.94–0.98) in the three measurement positions, namely I test, Y test, and T test [13]. Our data confirmed the results of the Ashworth et al. (2018) study, supporting the use of the ASH test together with a strength measurement device as a reliable tool for quantifying the ability to produce and transfer force across the shoulder girdle [13].

Reliability is defined as the extent to which measurements can be replicated [18]. In the past, paired *t*-tests, Bland–Altman plots, and Pearson correlation coefficient have been used to assess reliability. Today we know that these are not the best methods for evaluating reliability, as they only measure correlation [19]. In contrast, the ICC reflects both degrees of correlation and agreement between measurements, making it a better method for reliability assessment purposes [18]. Therefore, it has been widely used to evaluate inter-rater reliability, intrarater reliability, and test–retest reliability of, for example, different components of functional diagnostics [18,39,40,41]. The results from our study demonstrated good to excellent reliability of Active5™ for shoulder strength measurement at all three positions of the ASH test. Moreover, we observed excellent reliability for K-Force Plates for both inter-rater and test–retest reliability in dominant and nondominant limbs. However, the high repeatability obtained on both devices does not directly suggest that they may be used equally in assessing shoulder strength. As far as the values obtained at 180° and 90° using both studied devices were comparable, no correlation was found between the results gathered at 135°.

One group of authors reported lower reliability at the I test position [25], but the reason for this is unclear. They suggested this be caused by the highest forces typically occurring in the I test position, and therefore any variation potentially being more pronounced. This group used a sphygmomanometer for shoulder strength assessment, which has been reported to be less sensitive than a force plate in the ASH test. However, the results from our study did not support this idea, indicating a similar reliability level at all three positions.

Active5™ is a portable isometric-based strength training device that measures maximal isometric strength. So far, there is no literature available on the reliability and validity of the strength measurement using this device. The ASH test is simple to perform and interpret and does not require a lot of space. This facts in combination with a relatively inexpensive and easy-to-use device like Active5™ makes the test performance commonly available. The test may help to identify athletes with unknown conditions even if they do not present any signs or symptoms. The ASH test itself will not diagnose a problem affecting the strength of the shoulder and upper limb, but may indicate that one exists.

When performing between-group comparison and comparison of results at different time points, it must be remembered that obtained force values need to be normalized. Originally, Ashworth et al. (2018) normalized obtained values by dividing them by arm length value measured from the posterior angle of the acromion to the wrist joint line in each upper limb [13]. However, it remains unknown whether this kind of normalization is ideal as, for example, there have been no studies regarding the impact of body mass on the result of the ASH test.

As between-sex differences are considered highly important in the reliability of specific tests, the results in the present manuscript were analyzed separately for a group of females and a group of males. The reliability of the ASH test performed using Active5™ was comparable in both studied groups, indicating that it might be used for both women and men.

The main limitations of this study and, therefore, the validity of the test are the inclusion of healthy, recreational athletes without shoulder injuries only. Future research should focus on implementing the ASH test in both sports and clinical practice to determine its usefulness as a diagnostic and monitoring tool. Additionally, further study should be developed in terms of comparative analysis of ASH test results with other tools being used in upper limb examination.

## 5. Conclusions

The present study supports the isometric-based strength training device Active5™ as a reliable tool for the ASH test performance in females and males. Using a portable isometric-based strength training device, Active5™ constitutes a less expensive and more portable alternative to a gold standard force plate for ASH test purposes. It should be highlighted that the force values obtained with one of the studied devices can be compared to those obtained with the other device for measurements at 180° and 90° shoulder abduction. However, the comparison of results obtained at the angle of 135° should be carried out with considerable caution.

## Figures and Tables

**Table 1:** Illustration of consecutive tests being performed.

Active5™	Consecutive Tests	K-Force Plate	Consecutive Tests
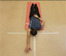	180°; I test	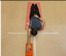	180°; I test
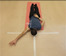	135°; Y test	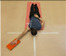	135°; Y test
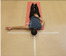	90°, T test	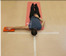	90°; T test

**Table 2:** Intrarater reliability of the ASH test in the female group using both studied devices.

Studied Limb	Shoulder Abduction	Active5™	K-Force Plates
Dominant limb	180°	0.932 (0.883, 0.960)	0.963 (0.933, 0.979)
	135°	0.959 (0.929, 0.976)	0.979 (0.963, 0.988)
	90°	0.941 (0.899, 0.966)	0.979 (0.964, 0.988)
Nondominant limb	180°	0.948 (0.908, 0.971)	0.979 (0.963, 0.988)
	135°	0.866 (0.776, 0.921)	0.982 (0.969, 0.990)
	90°	0.942 (0.900, 0.966)	0.959 (0.929, 0.976)

Values expressed as intraclass correlation coefficient (ICC) and 95% confidence interval (lower bound, upper bound) of the ICC.

**Table 3:** Inter-rater reliability of the ASH test in the female group using both studied devices.

Studied Limb	Shoulder Abduction	Active5™	K-Force Plates
Dominant limb	180°	0.969 (0.946, 0.982)	0.976 (0.956, 0.986)
	135°	0.967 (0.942, 0.981)	0.983 (0.970, 0.990)
	90°	0.976 (0.955, 0.985)	0.984 (0.972, 0.991)
Nondominant limb	180°	0.947 (0.904, 0.970)	0.968 (0.937, 0.983)
	135°	0.955 (0.922, 0.974)	0.983 (0.970, 0.990)
	90°	0.962 (0.935, 0.978)	0.981 (0.966, 0.989)

Values expressed as intraclass correlation coefficient (ICC) and 95% confidence interval (lower bound, upper bound) of the ICC.

**Table 4:** The results of force measurements at 180° shoulder abduction using Active5™ and K-Force Plates.

Force Measured at 180° Shoulder Abduction in Females (kg)							
Studied Limb	Session	Device	x	SD	CV %	SEM	MDC
Dominant	Session 1	Active5™	5.67	1.68	29.65	0.34	0.94
		K-Force Plates	5.37	1.63	30.37	0.33	0.91
	Session 2	Active5™	5.56	1.61	28.91	0.33	0.91
		K-Force Plates	5.23	1.65	31.53	0.34	0.94
	Session 3	Active5™	5.37	1.51	28.16	0.31	0.85
		K-Force Plates	5.32	1.64	30.94	0.34	0.94
Nondominant	Session 1	Active5™	4.95	1.60	32.29	0.32	0.88
		K-Force Plates	4.66	1.68	36.15	0.34	0.94
	Session 2	Active5™	4.75	1.60	33.59	0.32	0.88
		K-Force Plates	4.46	1.54	34.58	0.32	0.88
	Session 3	Active5™	4.86	1.64	33.89	0.34	0.94
		K-Force Plates	4.50	1.72	38.14	0.35	0.96

Values are expressed as arithmetic mean (x), standard deviation (SD), coefficient of variation (CV), and standard error of measurement (SEM); minimal detectable change (MDC).

**Table 5:** Correlation between ASH test results using Active5™ and K-Force plates in the dominant upper limb in the female group.

Correlation between ASH Test Results Using Active5™ and K-Force Plates in the Dominant Limb in Females				
		*p* Value	*r* Value	*r*^2^ Value
Session 1	180°	<0.0001	0.875	0.766
	135°	0.625	−0.104	0.010
	90°	<0.0001	0.931	0.868
Session 2	180°	<0.0001	0.879	0.773
	135°	0.591	−0.115	0.013
	90°	<0.0001	0.945	0.893
Session 3	180°	<0.0001	0.850	0.722
	135°	0.710	−0.079	0.001
	90°	<0.0001	0.919	0.845

Values expressed as a *p* value of statistical significance; *r* value: Pearson’s correlation coefficient and *r*^2^ indicating the proportion of variance.

**Table 6:** Correlation between ASH test results using Active5™ and K-Force plates in the nondominant upper limb in the female group.

Correlation between ASH Test Results Using Active5™ and K-Force Plates in the Nondominant Limb in Females				
		*p* Value	*r* Value	*r*^2^ Value
Session 1	180°	<0.0001	0.855	0.731
	135°	0.580	−0.118	0.014
	90°	<0.0001	0.862	0.744
Session 2	180°	<0.0001	0.865	0.749
	135°	0.795	−0.055	0.003
	90°	<0.0001	0.905	0.819
Session 3	180°	<0.0001	0.840	0.706
	135°	0.726	−0.075	0.005
	90°	<0.0001	0.888	0.789

Values expressed as a *p* value of statistical significance; *r* value: Pearson’s correlation coefficient and *r*^2^ indicating the proportion of variance.

**Table 7:** The results of force measurements at 135° shoulder abduction using Active5™ and K-Force Plates in the female group.

Force Measured at 135° Shoulder Abduction in Females (kg)							
Studied Limb	Session	Device	x	SD	CV %	SEM	MDC
Dominant	Session 1	Active5™	5.99	2.03	34.00	0.41	1.14
		K-Force Plates	7.12	3.26	45.79	0.66	1.82
	Session 2	Active5™	5.95	2.03	34.17	0.41	1.13
		K-Force Plates	7.15	3.16	44.29	0.64	1.77
	Session 3	Active5™	6.11	2.26	37.11	0.46	1.27
		K-Force Plates	7.29	3.28	45.01	0.66	1.82
Nondominant	Session 1	Active5™	5.50	2.04	37.06	0.41	1.13
		K-Force Plates	6.46	3.17	49.16	0.64	1.77
	Session 2	Active5™	5.42	1.98	36.61	0.40	1.11
		K-Force Plates	6.41	3.10	48.34	0.63	1.74
	Session 3	Active5™	5.48	2.14	39.11	0.43	1.19
		K-Force Plates	6.59	3.16	47.99	0.64	1.77

Values are expressed as arithmetic mean (x), standard deviation (SD), coefficient of variation (CV), and standard error of measurement (SEM); minimal detectable change (MDC).

**Table 8:** The results of force measurements at 90° shoulder abduction using Active5™ and K-Force Plates in the female group.

Force Measured at 90° of Shoulder Abduction in Females (kg)							
Studied Limb	Session	Device	x	SD	CV %	SEM	MDC
Dominant	Session 1	Active5™	6.88	2.99	43.50	0.61	1.69
		K-Force Plates	6.59	3.17	48.20	0.64	1.77
	Session 2	Active5™	6.69	3.00	44.80	0.61	1.68
		K-Force Plates	6.51	3.27	50.23	0.66	1.82
	Session 3	Active5™	6.96	2.91	41.94	0.59	1.63
		K-Force Plates	6.51	3.29	50.56	0.67	1.85
Nondominant	Session 1	Active5™	6.09	2.39	39.25	0.48	1.32
		K-Force Plates	5.62	2.36	41.97	0.48	1.32
	Session 2	Active5™	5.95	2.40	40.39	0.49	1.35
		K-Force Plates	5.61	2.37	42.40	0.48	1.32
	Session 3	Active5™	6.22	2.35	37.90	0.48	1.32
		K-Force Plates	5.55	2.42	43.71	0.49	1.35

Values are expressed as arithmetic mean (x), standard deviation (SD), coefficient of variation (CV), and standard error of measurement (SEM); minimal detectable change (MDC).

**Table 9:** Intrarater reliability of the ASH test using both studied devices in the male group.

Studied Limb	Shoulder Abduction	Active5™	K-Force Plates
Dominant limb	180°	0.900 (0.794, 0.953)	0.936 (0.855, 0.972)
	135°	0.961 (0.917, 0.982)	0.968 (0.932, 0.985)
	90°	0.925 (0.842, 0.965)	0.982 (0.961, 0.992)
Nondominant limb	180°	0.912 (0.806, 0.960)	0.968 (0.931, 0.985)
	135°	0.891 (0.762, 0.950)	0.980 (0.958, 0.991)
	90°	0.945 (0.884, 0.974)	0.978 (0.952, 0.990)

Values expressed as intraclass correlation coefficient (ICC) and 95% confidence interval (lower bound, upper bound) of the ICC.

**Table 10:** Inter-rater reliability of the ASH test using both studied devices in the male group.

Studied Limb	Shoulder Abduction	Active5™	K-Force Plates
Dominant limb	180°	0.949 (0.892, 0.976)	0.959 (0.912, 0.981)
	135°	0.972 (0.940, 0.987)	0.964 (0.924, 0.984)
	90°	0.970 (0.935, 0.986)	0.990 (0.979, 0.989)
Nondominant limb	180°	0.903 (0.796, 0.955)	0.968 (0.931, 0.985)
	135°	0.954 (0.902, 0.979)	0.954 (0.902, 0.979)
	90°	0.965 (0.925, 0.984)	0.989 (0.975, 0.995)

Values expressed as intraclass correlation coefficient (ICC) and 95% confidence interval (lower bound, upper bound) of the ICC.

**Table 11:** The results of force measurements at 180° shoulder abduction using Active5™ and K-Force Plates.

Force Measured at 180° of Shoulder Abduction in Males (kg)							
Studied Limb	Session	Device	x	SD	CV %	SEM	MDC
Dominant	Session 1	Active5™	9.48	3.00	31.65	0.57	1.57
		K-Force Plates	9.18	3.39	36.95	0.65	1.80
	Session 2	Active5™	9.33	2.95	31.65	0.56	1.55
		K-Force Plates	8.90	2.99	33.65	0.57	1.57
	Session 3	Active5™	9.28	3.43	36.98	0.66	1.82
		K-Force Plates	8.74	2.90	33.25	0.55	1.52
Nondominant	Session 1	Active5™	8.49	2.75	32.44	0.53	1.46
		K-Force Plates	7.72	2.90	37.57	0.55	1.52
	Session 2	Active5™	8.12	2.50	30.82	0.48	1.32
		K-Force Plates	7.40	2.61	35.25	0.50	1.38
	Session 3	Active5™	8.05	2.65	32.92	0.51	1.41
		K-Force Plates	7.62	2.70	35.53	0.52	1.44

Values are expressed as arithmetic mean (x), standard deviation (SD), coefficient of variation (CV), and standard error of measurement (SEM); minimal detectable change (MDC).

**Table 12:** Correlation between ASH test results using Active5™ and K-Force plates in the dominant upper limb in the male group.

Correlation between ASH Test Results Using Active5™ and K-Force Plates in the Dominant Limb in Males				
		*p* Value	*r* Value	*r*^2^ Value
Session 1	180°	<0.0001	0.743	0.552
	135°	0.170	0.272	0.074
	90°	<0.0001	0.682	0.465
Session 2	180°	<0.0001	0.855	0.731
	135°	0.164	0.275	0.076
	90°	<0.0001	0.666	0.443
Session 3	180°	<0.0001	0.846	0.716
	135°	0.064	0.362	0.131
	90°	<0.0001	0.698	0.487

Values expressed as a *p* value of statistical significance; *r* value: Pearson’s correlation coefficient and *r*^2^ indicating the proportion of variance.

**Table 13:** Correlation between ASH test results using Active5™ and K-Force plates in the nondominant upper limb in the male group.

Correlation between ASH Test Results Using Active5™ and K-Force Plates in the Nondominant Limb in Males				
		*p* Value	*r* Value	*r*^2^ Value
Session 1	180°	<0.0001	0.791	0.626
	135°	0.152	0.283	0.080
	90°	<0.0001	0.642	0.412
Session 2	180°	<0.0001	0.788	0.621
	135°	0.097	0.326	0.106
	90°	0.001	0.618	0.382
Session 3	180°	<0.0001	0.865	0.748
	135°	0.147	0.286	0.082
	90°	<0.0001	0.707	0.499

Values expressed as a *p* value of statistical significance; *r* value: Pearson’s correlation coefficient and *r*^2^ indicating the proportion of variance.

**Table 14:** The results of force measurements at 135° of shoulder abduction using Active5™ and K-Force Plates.

Force Measured at 135° of Shoulder Abduction in Males (kg)							
Studied Limb	Session	Device	x	SD	CV %	SEM	MDC
Dominant	Session 1	Active5™	7.65	2.95	38.62	0.56	1.55
		K-Force Plates	5.76	1.84	31.96	0.35	0.96
	Session 2	Active5™	7.54	3.05	40.48	0.58	1.60
		K-Force Plates	5.83	1.84	31.67	0.35	0.96
	Session 3	Active5™	7.61	3.26	42.79	0.62	1.71
		K-Force Plates	5.87	1.91	32.51	0.36	0.99
Nondominant	Session 1	Active5™	6.75	2.34	34.66	0.45	1.24
		K-Force Plates	5.12	1.75	34.25	0.33	0.91
	Session 2	Active5™	6.87	2.75	40.10	0.53	1.46
		K-Force Plates	5.11	1.65	32.35	0.31	0.85
	Session 3	Active5™	6.55	2.86	43.63	0.55	1.52
		K-Force Plates	5.19	1.74	33.49	0.33	0.91

Values are expressed as arithmetic mean (x), standard deviation (SD), coefficient of variation (CV), and standard error of measurement (SEM); minimal detectable change (MDC).

**Table 15:** The results of force measurements at 135° of shoulder abduction using Active5™ and K-Force Plates.

Force Measured at 90° of Shoulder Abduction in Males (kg)							
Studied Limb	Session	Device	x	SD	CV %	SEM	MDC
Dominant	Session 1	Active5™	5.95	2.04	34.39	0.39	1.08
		K-Force Plates	5.37	1.71	31.87	0.32	0.88
	Session 2	Active5™	5.95	2.11	35.60	0.40	1.11
		K-Force Plates	5.33	1.75	32.88	0.33	0.91
	Session 3	Active5™	6.05	2.31	38.18	0.44	1.21
		K-Force Plates	5.41	1.71	31.60	0.32	0.88
Nondominant	Session 1	Active5™	5.36	1.65	30.87	0.31	0.85
		K-Force Plates	4.76	1.34	28.30	0.25	0.69
	Session 2	Active5™	5.41	1.88	34.80	0.36	0.99
		K-Force Plates	4.75	1.41	29.67	0.27	0.74
	Session 3	Active5™	5.46	1.89	34.75	0.36	0.99
		K-Force Plates	4.77	1.34	28.11	0.25	0.69

Values are expressed as arithmetic mean (x), standard deviation (SD), coefficient of variation (CV), and standard error of measurement (SEM); minimal detectable change (MDC).

## Data Availability

The datasets generated and analyzed during the current study are available from the corresponding author on reasonable request.

## Supplementary Materials

The following supporting information can be downloaded at: https://www.mdpi.com/article/10.3390/biology11040577/s1, Supplementary Material S1: Figure S1–S4. Bland–Altman plots comparing the obtained values of force measured during the three sessions by two raters in three different angular positions in dominant and nondominant shoulders using Active5™ and K-Force Plates.

## References

[1] Torres-Banduc M.A., Jerez-Mayorga D., Moran J., Keogh J.W.L., Ramírez-Campillo R. (2021). Isokinetic force-power profile of the shoulder joint in males participating in CrossFit training and competing at different levels. PeerJ.

[2] Kim B.G., Lim S.K., Kong S. (2021). The Relationship between Scapular Upward Rotation and Shoulder Internal and External Rotation Isokinetic Strength in Professional Baseball Pitchers. Healthcare.

[3] Vargas V.Z., Motta C., Vancini R.L., de Lira C.A.B., Andrade M.S. (2021). Shoulder Isokinetic Strength Balance Ratio in Overhead Athletes: A Cross-Sectional Study. Int. J. Sports Phys. Ther..

[4] Lucena E.G., Ferland P.M., Ahmadi S., Teixeira L.F., Comtois A.S., Uchida M.C. (2022). Isokinetic strength of shoulder rotator muscles in powerlifters: Correlation between isometric and concentric muscle actions. J. Sports Med. Phys. Fit..

[5] Chamorro C., Arancibia M., Trigo B., Arias-Poblete L., Jerez-Mayorga D. (2021). Absolute Reliability and Concurrent Validity of Hand-Held Dynamometry in Shoulder Rotator Strength Assessment: Systematic Review and Meta-Analysis. Int. J. Environ. Res. Public Health.

[6] Karabay D., Yesilyaprak S.S., Sahiner Picak G. (2020). Reliability and validity of eccentric strength measurement of the shoulder abductor muscles using a hand-held dynamometer. Phys. Ther. Sport.

[7] Fieseler G., Molitor T., Irlenbusch L., Delank K.S., Laudner K.G., Hermassi S., Schwesig R. (2015). Intrarater reliability of goniometry and hand-held dynamometry for shoulder and elbow examinations in female team handball athletes and asymptomatic volunteers. Arch. Orthop. Trauma Surg..

[8] Cools A.M., De Wilde L., Van Tongel A., Ceyssens C., Ryckewaert R., Cambier D.C. (2014). Measuring shoulder external and internal rotation strength and range of motion: Comprehensive intra-rater and inter-rater reliability study of several testing protocols. J. Shoulder Elbow Surg..

[9] Hirano M., Katoh M. (2015). Absolute reliability of shoulder joint horizontal adductor muscle strength measurements using a handheld dynamometer. J. Phys. Ther. Sci..

[10] Katoh M. (2015). Test-retest reliability of isometric shoulder muscle strength measurement with a handheld dynamometer and belt. J. Phys. Ther. Sci..

[11] Herrington L., Horsley I., Rolf C. (2010). Evaluation of shoulder joint position sense in both asymptomatic and rehabilitated professional rugby players and matched controls. Phys. Ther. Sport.

[12] Croteau F., Robbins S.M., Pearsall D. (2021). Hand-Held Shoulder Strength Measures Correlate With Isokinetic Dynamometry in Elite Water Polo Players. J. Sport Rehabil..

[13] Ashworth B., Hogben P., Singh N., Tulloch L., Cohen D.D. (2018). The Athletic Shoulder (ASH) test: Reliability of a novel upper body isometric strength test in elite rugby players. BMJ Open Sport Exerc. Med..

[14] Awatani T., Mori S., Shinohara J., Koshiba H., Nariai M., Tatsumi Y., Nagata A., Morikita I. (2016). Same-session and between-day intra-rater reliability of hand-held dynamometer measurements of isometric shoulder extensor strength. J. Phys. Ther. Sci..

[15] Impellizzeri F.M., Marcora S.M. (2009). Test validation in sport physiology: Lessons learned from clinimetrics. Int. J. Sports Physiol. Perform..

[16] Bujang M.A., Baharum N. (2017). A simplified guide to determination of sample size requirements for estimating the value of intraclass correlation coefficient: A review. Arch. Orofac. Sci..

[17] Shrout P.E., Fleiss J.L. (1979). Intraclass correlations: Uses in assessing rater reliability. Psychol. Bull..

[18] Koo T.K., Li M.Y. (2016). A Guideline of Selecting and Reporting Intraclass Correlation Coefficients for Reliability Research. J. Chiropr. Med..

[19] Hopkins W.G. (2000). Measures of reliability in sports medicine and science. Sports Med..

[20] De la Torre J., Marin J., Polo M., Marín J.J. (2020). Applying the Minimal Detectable Change of a Static and Dynamic Balance Test Using a Portable Stabilometric Platform to Individually Assess Patients with Balance Disorders. Healthcare.

[21] Bland J.M., Altman D.G. (1996). Measurement error proportional to the mean. BMJ.

[22] Mukaka M.M. (2012). Statistics corner: A guide to appropriate use of correlation coefficient in medical research. Malawi Med. J..

[23] Headey J., Brooks J.H., Kemp S.P. (2007). The epidemiology of shoulder injuries in English professional rugby union. Am. J. Sports Med..

[24] Yeomans C., Kenny I.C., Cahalan R., Warrington G.D., Harrison A.J., Hayes K., Lyons M., Campbell M.J., Comyns T.M. (2018). The Incidence of Injury in Amateur Male Rugby Union: A Systematic Review and Meta-Analysis. Sports Med..

[25] Morrison G., Ashworth B., Taylor-Kaveney T. (2021). The validity of the sphygmomanometer for shoulder strength assessment in amateur rugby union players. Phys. Ther. Sport.

[26] Crichton J., Jones D.R., Funk L. (2012). Mechanisms of traumatic shoulder injury in elite rugby players. Br. J. Sports Med..

[27] Fuller C.W., Taylor A., Kemp S.P., Raftery M. (2017). Rugby World Cup 2015: World Rugby injury surveillance study. Br. J. Sports Med..

[28] Hodder J.N., Keir P.J. (2012). Targeted gripping reduces shoulder muscle activity and variability. J. Electromyogr. Kinesiol..

[29] Martin H.J., Yule V., Syddall H.E., Dennison E.M., Cooper C., Aihie Sayer A. (2006). Is hand-held dynamometry useful for the measurement of quadriceps strength in older people? A comparison with the gold standard Bodex dynamometry. Gerontology.

[30] Bohannon R.W. (2005). Manual muscle testing: Does it meet the standards of an adequate screening test?. Clin. Rehabil..

[31] Aguiar L.T., Lara E.M., Martins J.C., Teixeira-Salmela L.F., Quintino L.F., Christo P.P. (2016). Modified sphygmomanometer test for the assessment of strength of the trunk, upper and lower limbs muscles in subjects with subacute stroke: Reliability and validity. Eur. J. Phys. Rehabil. Med..

[32] Delahunt E., McEntee B.L., Kennelly C., Green B.S., Coughlan G.F. (2011). Intrarater reliability of the adductor squeeze test in gaelic games athletes. J. Athl. Train..

[33] McCall A., Nedelec M., Carling C., Le Gall F., Berthoin S., Dupont G. (2015). Reliability and sensitivity of a simple isometric posterior lower limb muscle test in professional football players. J. Sports Sci..

[34] Verdera F., Champavier L., Schmidt C., Bermon S., Marconnet P. (1999). Reliability and validity of a new device to measure isometric strength in polyarticular exercises. J. Sports Med. Phys. Fit..

[35] Barbosa A.C., Intelangelo L., Bordachar D., Fernandes I., Cardoso D., Fernandes I., Porfírio W., Felício D. (2018). Validity and reliability of shoulder strength assessment during scaption, internal rotation and external rotation using an anchored, non-modified sphygmomanometer. Hum. Mov..

[36] Matthews M.J., Green D., Matthews H., Swanwick E. (2017). The effects of swimming fatigue on shoulder strength, range of motion, joint control, and performance in swimmers. Phys. Ther. Sport.

[37] Cadogan A., Laslett M., Hing W., McNair P., Williams M. (2011). Reliability of a new hand-held dynamometer in measuring shoulder range of motion and strength. Man Ther..

[38] Holt K.L., Raper D.P., Boettcher C.E., Waddington G.S., Drew M.K. (2016). Hand-held dynamometry strength measures for internal and external rotation demonstrate superior reliability, lower minimal detectable change and higher correlation to isokinetic dynamometry than externally-fixed dynamometry of the shoulder. Phys. Ther. Sport.

[39] Czamara A., Królikowska A., Szuba Ł., Widuchowski W., Kentel M. (2015). Single- vs. double-bundle anterior cruciate ligament reconstruction: A new aspect of knee assessment during activities involving dynamic knee rotation. J. Strength Cond. Res..

[40] Królikowska A., Czamara A., Szuba Ł., Reichert P. (2018). The Effect of Longer versus Shorter Duration of Supervised Physiotherapy after ACL Reconstruction on the Vertical Jump Landing Limb Symmetry. BioMed Res. Int..

[41] Królikowska A., Czamara A., Kentel M. (2015). Does Gracilis Tendon Harvest During ACL Reconstruction with a Hamstring Autograft Affect Torque of Muscles Responsible for Shin Rotation?. Med. Sci. Monit..

