# Supplementary material for: Reliability and Validity of the Athletic Shoulder (ASH) Test Performed Using Portable Isometric-Based Strength Training Device
Source: Biology (Basel). 2022 Apr 11;11(4):577. doi: 10.3390/biology11040577 (PMC9024613; doi:10.3390/biology11040577)
Supplement: Supplementary file 1 [file biology-11-00577-s001.zip › biology-1650645-SI.pdf]

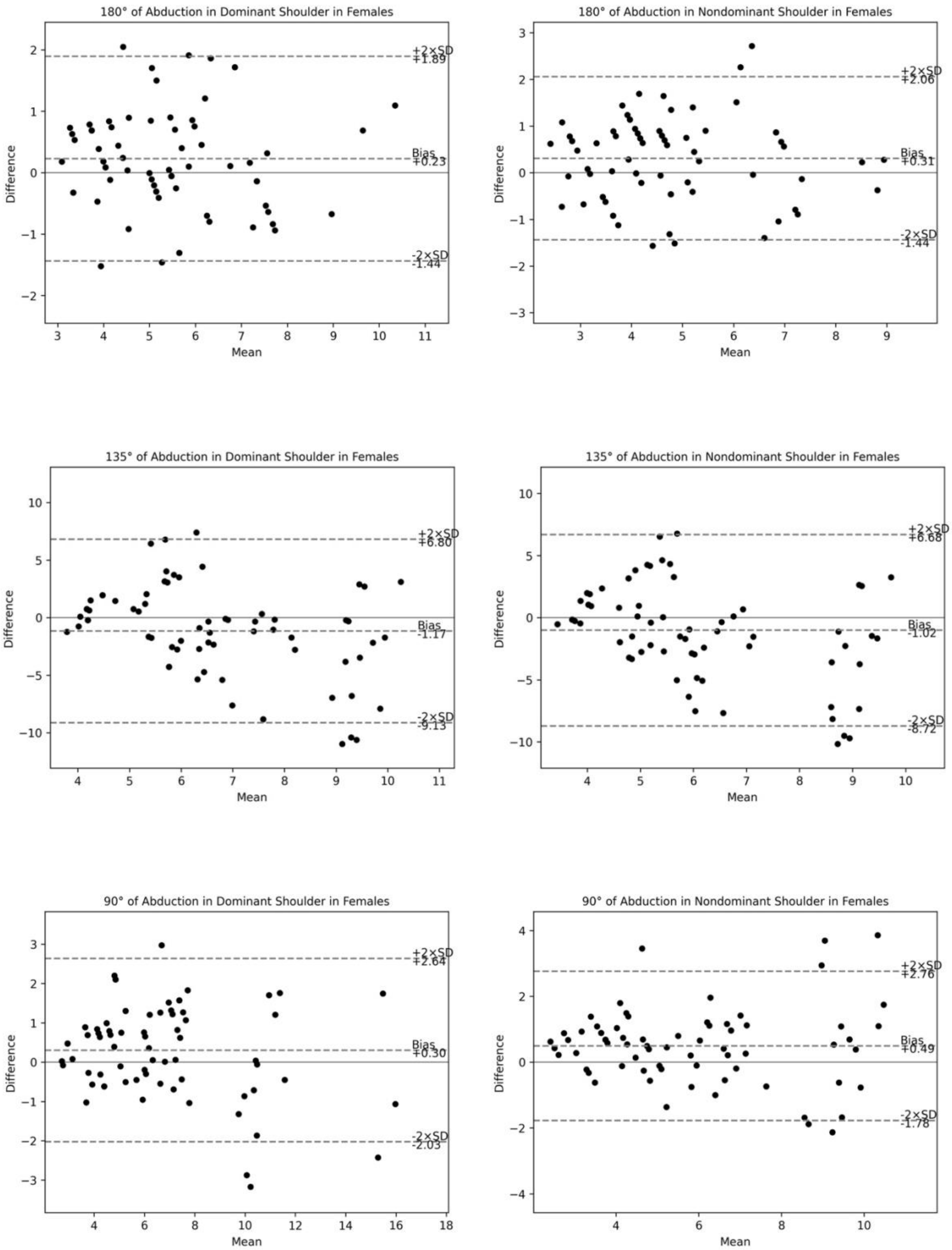

Figure S1. Bland-Altman plots comparing the obtained values of force measured during the three sessions by two raters in three different angular positions in dominant and nondominant shoulders using Active5™ and K-Force Plates in females.

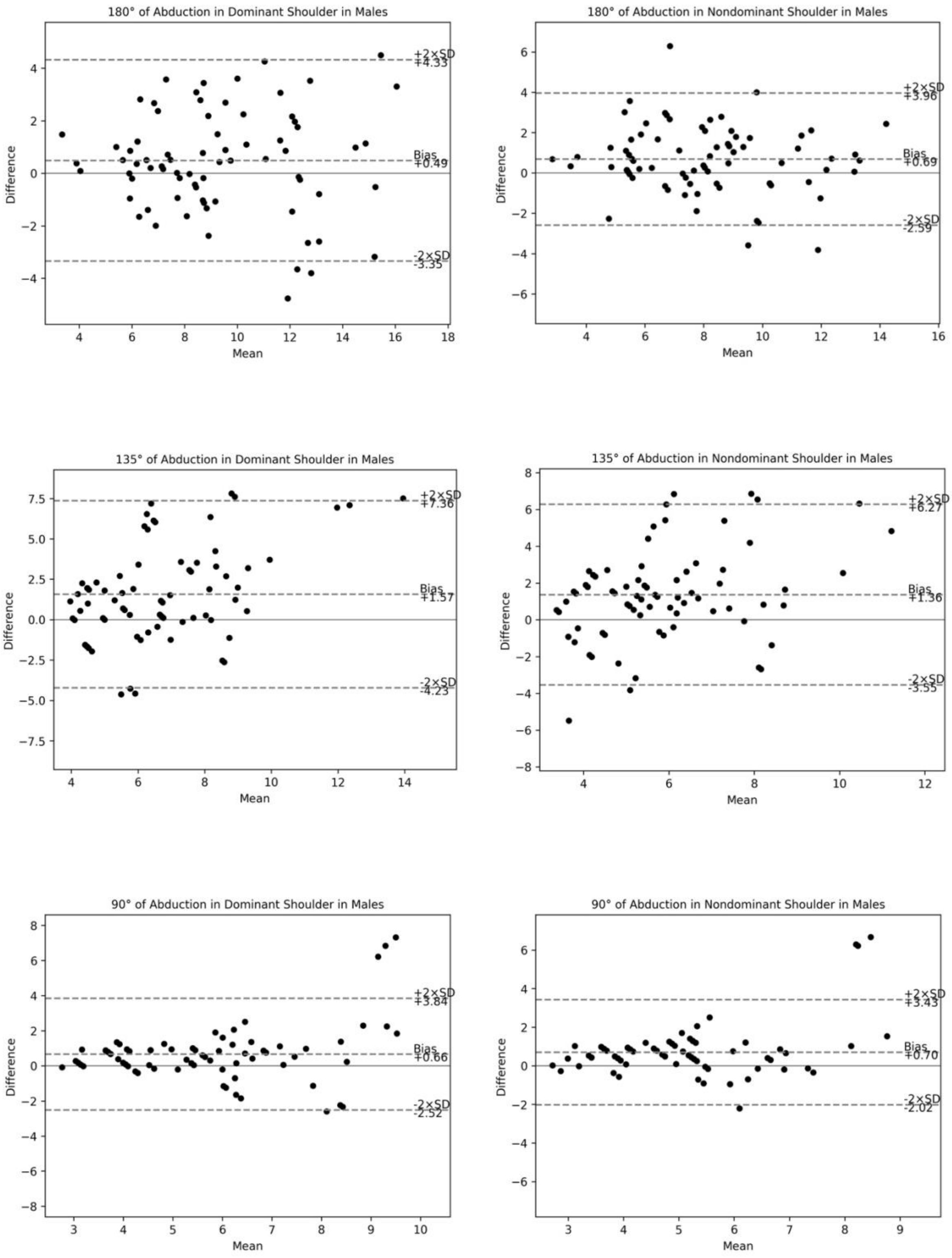

Figure S2. Bland-Altman plots comparing the obtained values of force measured during the three sessions by two raters in three different angular positions in dominant and nondominant shoulders using Active5™ and K-Force Plates in males.

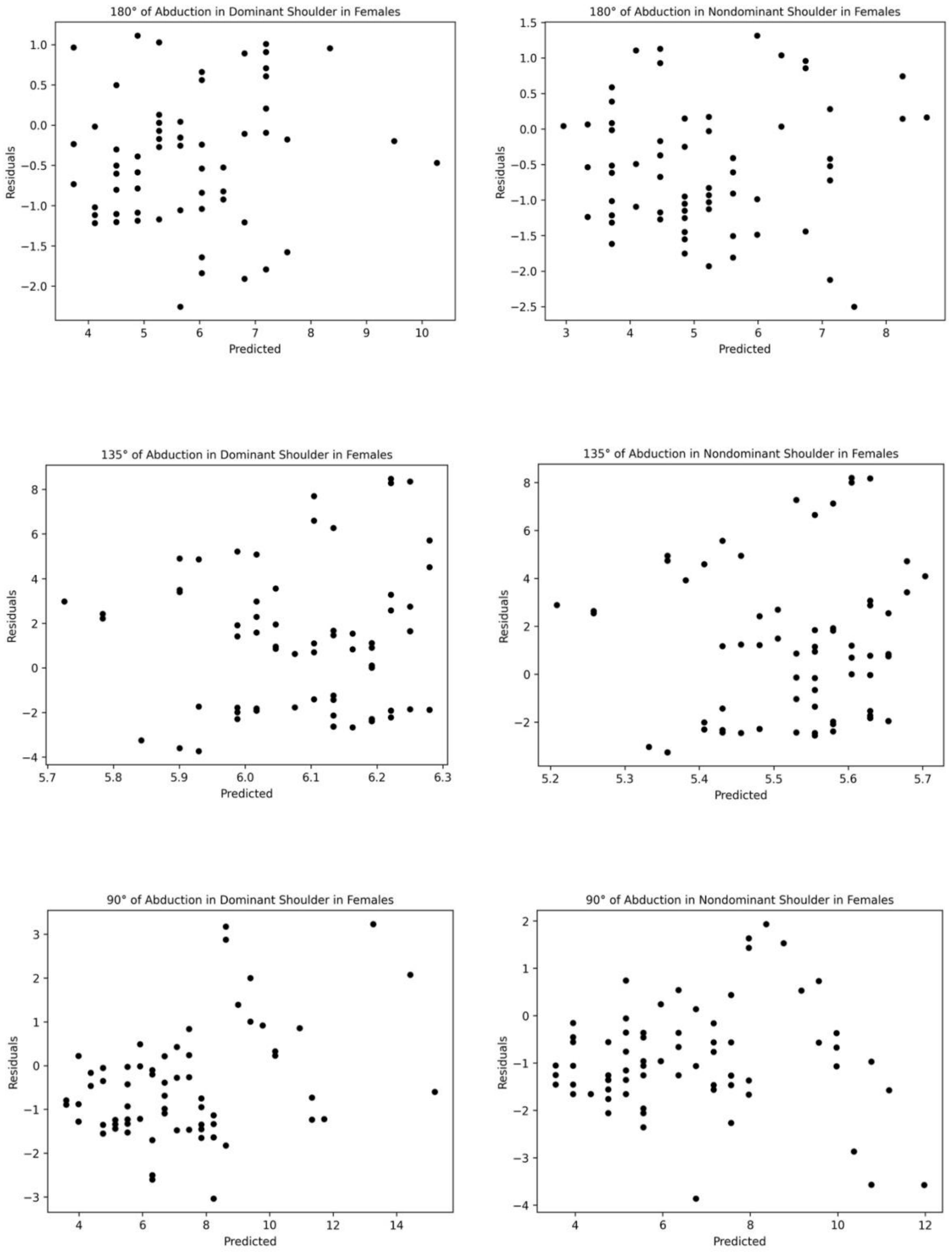

Figure S3 Plots comparing residuals and predicted values of force measured during the three sessions by two raters in three different angular positions in dominant and nondominant shoulders using Active5™ and K-Force Plates in females.

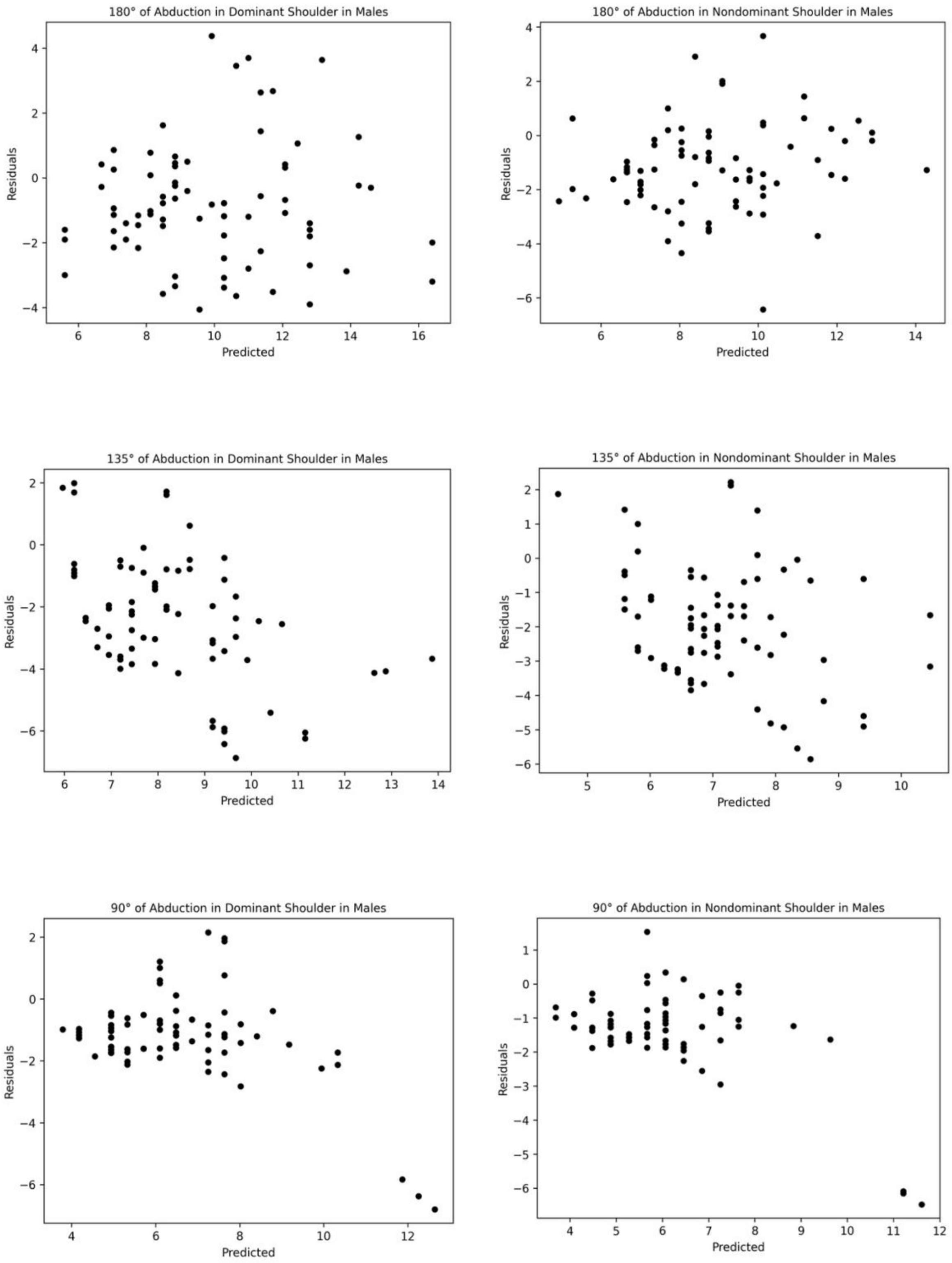

Figure S4. Plots comparing residuals and predicted values of force measured during the three sessions by two raters in three different angular positions in dominant and nondominant shoulders using Active5™ and K-Force Plates in males.
